# NDUFAF7 Methylates Arginine 85 in the NDUFS2 Subunit of Human Complex I*

**DOI:** 10.1074/jbc.M113.518803

**Published:** 2013-10-02

**Authors:** Virginie F. Rhein, Joe Carroll, Shujing Ding, Ian M. Fearnley, John E. Walker

**Affiliations:** From the Medical Research Council Mitochondrial Biology Unit, Cambridge CB2 0XY, United Kingdom

**Keywords:** Bioenergetics, Electron Transport, Mitochondria, Protein Methylation, Respiratory Chain, Assembly, Complex I, Dimethylarginine, Methyltransferase

## Abstract

Complex I (NADH ubiquinone oxidoreductase) in mammalian mitochondria is an L-shaped assembly of 44 subunits. One arm is embedded in the inner membrane with the other protruding ∼100 Å into the matrix of the organelle. The extrinsic arm contains binding sites for NADH and the primary electron acceptor FMN, and it provides a scaffold for seven iron-sulfur clusters that form an electron pathway linking FMN to the terminal electron acceptor, ubiquinone, which is bound in the region of the junction between the arms. The membrane arm contains four antiporter-like domains, probably energetically coupled to the quinone site and involved in pumping protons from the matrix into the intermembrane space contributing to the proton motive force. Complex I is put together from preassembled subcomplexes. Their compositions have been characterized partially, and at least 12 extrinsic assembly factor proteins are required for the assembly of the complex. One such factor, NDUFAF7, is predicted to belong to the family of *S*-adenosylmethionine-dependent methyltransferases characterized by the presence in their structures of a seven-β-strand protein fold. In the present study, the presence of NDUFAF7 in the mitochondrial matrix has been confirmed, and it has been demonstrated that it is a protein methylase that symmetrically dimethylates the ω-*N*^G^,*N*^G′^ atoms of residue Arg-85 in the NDUFS2 subunit of complex I. This methylation step occurs early in the assembly of complex I and probably stabilizes a 400-kDa subcomplex that forms the initial nucleus of the peripheral arm and its juncture with the membrane arm.

## Teaser

**Background:** Arginine 85 of NDUFS2, a subunit of mitochondrial complex I, is symmetrically dimethylated.

**Results:** NDUFAF7, a protein methylase in the matrix of mitochondria, modifies arginine 85 of NDUFS2 during assembly of complex I.

**Conclusion:** Methylation of arginine 85 of NDUFS2 is required for assembly of complex I.

**Significance:** The arginine protein methylase, NDUFAF7, is an assembly factor for human complex I.

## Introduction

Mammalian complex I (NADH:ubiquinone oxidoreductase) is an L-shaped protein complex of 44 subunits with a combined molecular mass of ∼1 MDa bound in the inner membranes of mitochondria (1–3). One arm of the complex extends into the matrix of the organelle, and the orthogonal arm is bound in the inner membrane (4). The extrinsic arm contains the binding sites for NADH and the primary electron acceptor, FMN, and also a chain of seven iron-sulfur clusters that carries the electrons to the terminal acceptor, coenzyme Q. The oxidized form of coenzyme Q binds in the region of the juncture between the extrinsic and membrane arms (5, 6). Once reduced, it is released into the reduced quinone pool from where it carries the electrons to the cytochrome *bc*_1_ complex. The transfer of each two electrons through complex I is coupled to the extrusion of four protons from the mitochondrial matrix into the intermembrane space (7, 8), probably via four antiporter folds in the membrane domain (6). Thus, complex I contributes to the generation of the transmembrane proton motive force, which is itself coupled energetically to ATP synthesis, metabolite transport, and other activities. Seven hydrophobic subunits, known as ND1–ND6 and ND4L, in the membrane arm that are encoded in mitochondrial DNA and seven of the nuclear encoded subunits in the peripheral arm together form the catalytic core of the enzyme. This catalytic core is conserved in complexes I in eukaryotes and eubacteria (9, 10). The remaining subunits are distributed between peripheral and membrane arms and do not evidently participate in the known catalytic activities of complex I. They are known collectively as the “supernumerary” subunits (9, 11). Some of them may have a role in the assembly of complex I (12–15).

Specific human pathogenic mutations have been mapped to all of the core subunits of complex I and currently to 10 of the supernumerary subunits (16–19). Frequently, they interfere with the assembly of complex I leading to deficiency of the active enzyme and often to the accumulation of subcomplexes of the enzyme that represent stalled intermediates in the pathway of assembly (16). The partial characterization of these subcomplexes provides the basis of a model for the assembly process in which two distinct preformed subassemblies are joined together to provide the initial junction between the membrane and matrix arms. This intermediate complex is expanded by the addition of the distal region of the membrane arm, a domain containing the NADH and FMN binding sites to the distal region of the matrix arm, and the remaining supernumerary subunits (16). This assembly process depends upon the participation of at least 12 exogenous protein factors that are not constituents of the mature functional enzyme. Six of them, NDUFAF1–4, ACAD9, and ECSIT, bind specifically to various subcomplexes in the assembly pathway (20–25), but their molecular functions and the roles of the six other factors NDUFAF5–7, FOXRED1, NUBPL, and TMEM126B (26–31) in complex I assembly are not known. Pathogenic human mutations have been mapped to *NDUFAF1–4*, *ACAD9*, *FOXRED1*, and *NUBPL* (20, 23, 25, 29, 32, 33). NUBPL may be required for the process of incorporation of iron-sulfur clusters into subunits of complex I (28). NDUFAF5 and 6 appear to stabilize by unknown mechanisms the initial nucleating subcomplex of the matrix arm that interacts with the membrane subunit ND1 (34, 35). Mutations in NDUFAF5 and 6, or suppression of expression of NDUFAF7, affect its assembly and impair its activity (26, 27, 30). NDUFAF6 could be a phytoene synthase involved in biosynthesis of carotenoids, and NDUFAF5 and NDUFAF7 are predicted to belong to the family of *S*-adenosylmethionine-dependent methyltransferases characterized by the presence in their structures of a 7β-strand protein fold (36). It has been suggested from indirect evidence obtained in *Dictyostelium* that MidA, the orthologue of NDUFAF7, might be a protein methylase, and mutation of specific conserved residues that have been associated with the enzymic mechanism of methylases supported this suggestion (30). These residues are conserved in the human orthologue. Based on yeast two-hybrid screens, evidence was provided that *Dictyostelium* MidA interacts with the NDUFS2 (49 kDa) subunit of *Dictyostelium* complex I. Moreover, on the basis of proteomic studies conducted in rat that proposed that Arg-290 of the NDUFS2 subunit is monomethylated, it was suggested that the orthologous residue in human subunit NDUFS2 might be the residue methylated by human NDUFAF7 (30). However, there was no direct evidence from these earlier studies that human NDUFAF7 is a protein methylase or that it modifies by methylation any subunit of human complex I, including the NDUFS2 subunit. It is now known that Arg-290 is not methylated in either bovine or human mitochondria (37).

As reported below, we have confirmed a previous observation (30) that NDUFAF7 is targeted to human mitochondria. We have established that it is a protein methylase and that it is responsible for the methylation of residue Arg-85 in the NDUFS2 subunit of complex I. We have shown recently that this residue carries two methyl groups attached symmetrically to the ω-*N*^G^- and ω-*N*^G′^-nitrogen atoms of its guanidino group (37).

## EXPERIMENTAL PROCEDURES

### 

#### 

##### Bioinformatic Analyses

The 66 known and 142 putative human methyltransferases (36) were examined for the presence of N-terminal mitochondrial targeting sequences with the programs MitoProt, iPSORT, and TargetP (38–40).

##### Cell Culture

Human 143B osteosarcoma cells (ATCC number CRL8303) were grown at 37 °C in DMEM containing 25 mm glucose and supplemented with FBS (10% v/v), penicillin (100 units/ml), and streptomycin (0.1 mg/ml) under an atmosphere of 5% CO_2_.

##### Determination of the Subcellular Location of NDUFAF7

The cDNA for human NDUFAF7 (Fisher) was amplified with the forward and reverse primers 5′-GCCTGATCAGGAATGAGTGTACTGCTGAGGTCAG-3′ and 5′-ACCGCTCGAGCTGCCAAGCAAGTTCACTAAAC-3′, respectively, encoding FLAG and Strep II tags at the C terminus of the protein, and cloned into plasmid pcDNA^TM^5/FRT/TO (Invitrogen). The plasmid DNA was transfected into 143B cells with Lipofectamine 2000 (Invitrogen). After 24 h, the mitochondria were labeled with 200 nm MitoTracker Orange (Invitrogen) and nuclei with DAPI stain (0.4 μg/ml). The same cells were fixed with 4% (w/v) paraformaldehyde and permeabilized with Triton X-100 (0.5% v/v). The FLAG-tagged proteins were detected with mouse M2 anti-FLAG antibody (Sigma) followed by Alexa Fluor 488 goat anti-mouse secondary antibody (Invitrogen). The fluorescent signal was visualized with a Zeiss 510 LSM confocal microscope.

##### Suppression of Expression of Subunits of Complex I

Transcripts for NDUFAF7 were suppressed transiently in human 143B osteosarcoma cells with 30 nm siRNA (MISSION siRNA; Sigma). Three successive transfections were performed at 72-h intervals. A negative control siRNA (Allstars negative control siRNA; Qiagen) was used at the same concentration. Reduction of transcripts for NDUFAF7 was assessed by quantitative real time PCR performed with a TaqMan gene expression assay (Invitrogen), on cDNA prepared with a Cells-to-CT kit (Invitrogen). The levels of the transcripts for NDUFAF7 (normalized to endogenous β-actin) were investigated, and samples enriched for mitoplasts or inner mitochondrial membrane proteins were prepared 48 h after each transfection. The phenotype of cells was assessed 48 h after the second and third transfections.

##### Measurement of Respiration

The impact of the suppression of expression of NDUFAF7 on the OCR^3^ by human osteosarcoma 143B cells was measured in an XF24 extracellular flux analyzer (Seahorse Biosciences, North Billerica, MA) 48 h after the second and third transfections with siRNA at 120 and 192 h. The assay medium consisted of DMEM base formulation (Sigma) plus NaCl (1.85 g/liter), 2 mm glucose, 2 mm GlutaMAX (Invitrogen), 1 mm pyruvate, phenol red (15 mg/liter), and 20 mm HEPES (pH 7.4). Cells in assay medium were equilibrated for 1 h at 37 °C in air. Then basal OCR was measured and either 2-deoxyglucose (20 mm), rotenone (600 nm), duroquinol (627 μm), and antimycin (600 nm), or 2-deoxyglucose (20 mm), oligomycin (100 nm), carbonylcyanide *p-*(trifluoromethoxy)phenylhydrazone (500 nm), and a combination of rotenone (600 nm) and antimycin A (600 nm), were added sequentially. Complex I-dependent OCR was corrected for nonmitochondrial oxygen consumption by subtraction of rotenone-inhibited OCR values from values obtained after the addition of 2-deoxyglucose; complex III-dependent OCR was corrected for nonmitochondrial oxygen consumption by subtraction of antimycin A-inhibited OCR values from values obtained after the addition of duroquinol. Respiration was normalized to cell number by the sulforhodamine B colorimetric assay (41). *p* values for the OCR were calculated with a paired Student's *t* test.

##### Protein Analyses

Cells were suspended at a protein concentration of 10 mg/ml in PBS with complete EDTA-free mix of protease inhibitor (Roche Applied Science) and enriched for mitoplasts by addition of an equal volume of digitonin (1 mg/ml) in PBS inhibitor, to give a detergent:protein ratio of 1:10 (w/w) (42). The sample was centrifuged (11,000 × *g*, 5 min, 4 °C), and crude mitoplasts were solubilized from the pellet with 1% (w/v) *n*-dodecyl β-d-maltoside. Samples enriched for inner mitochondrial membrane proteins were prepared by using a higher concentration of digitonin (4 mg/ml). Proteins were fractionated by SDS-PAGE on Novex Tris-glycine 10–20% acrylamide gradient gels (Invitrogen) and transferred electrophoretically from gels to an Immobilon P membrane (Millipore, Billerica, MA) at 300 mA for 60 min in chilled buffer consisting of 10 mm sodium bicarbonate, 3 mm sodium carbonate, and 0.025% (w/v) SDS. The membrane was washed in PBS containing 0.01% (v/v) Tween 20 (PBST) and then blocked with a solution of dried skimmed milk in PBST. The treated membrane was incubated with primary antibodies and then with a goat anti-rabbit (Fisher Scientific) or rabbit anti-chicken (Sigma) peroxidase-conjugated secondary antibody. The antibody incubations were performed in milk-PBST. Primary rabbit antibodies against the complex I subunits NDUFS2 and NDUFS7 were obtained from Abcam (Cambridge, UK), and the rabbit anti-citrate synthase antibody was purchased from Proteintech (Manchester, UK). A primary chicken antibody against the human ND1 peptide AETNRTPFDLAEGE was produced by Agrisera (Vannas, Sweden). Bound antibodies were detected by chemiluminescence using ECL prime reagents (GE Healthcare).

To assess the impact of suppression of expression of NDUFAF7 on the assembly of complex I, samples enriched for inner mitochondrial membrane proteins were analyzed by blue native PAGE in bis-Tris 3–12% acrylamide gradient gels (Invitrogen; 75 V for 1 h and 150 V for 2 h at 4 °C). Fractionated proteins were transferred electrophoretically in transfer buffer lacking SDS to an Immobilon P membrane, as described above. Membranes were probed with antibodies against subunits NDUFB8 (Sigma), NDUFS2 of complex I, and the SDHB subunit of complex II (Sigma).

##### Mass Spectrometric Analysis

The effect of the suppression of expression of NDUFAF7 on the methylation status of NDUFS2 was studied by analyzing the two tryptic peptides (residues 75–89 and 76–89) containing the methylated arginine 85. Crude mitoplast proteins were reduced with 5 mm Tris (2-carboxyethyl)-phosphine, pH 7.0, alkylated with 15 mm iodoacetamide in SDS gel sample buffer at pH 8.0, and fractionated on Novex Tris-glycine 10–20% (Invitrogen) acrylamide gradient gels. Proteins were identified by tryptic mass mapping of Coomassie Blue-stained bands or sections of gel (43) in a MALDI-TOF-TOF mass spectrometer (model 4800; AB-Sciex, Warrington, UK) with α-cyano-4-hydroxycinnamic acid as matrix. CID was performed with air. Peptides were fractionated also by reverse phase nanochromatography with a Proxeon Easy-nLC instrument (Thermo Fisher, Hemel Hempstead, UK). The C_18_ column (75-μm inner diameter × 100 mm; Nanoseparations, Nieuwkoop, The Netherlands) was eluted with an acetonitrile gradient in 0.1% (v/v) formic acid at 250 nl/min and was coupled directly to an LTQ OrbiTrap XL-ETD (electron transfer dissociation) mass spectrometer (Thermo Fisher). Peptides were fragmented by CID with nitrogen. Proteins were identified from peptide mass and fragmentation data by interrogation of NCBInr protein sequence databases with Proteome Discoverer 1.3 (Thermo Fisher) and the Mascot search engine (44). The relative abundance of two methylated tryptic peptides (residues 76–89 and 75–89 of subunit NDUFS2; CamDPHIGLLHRGTEK and KCamDPHIGLLHRGTEK), plus one nonmethylated version (residues 75–85, KCamDPHIGLLHR) found only in cells where expression of NDUFAF7 had been suppressed, were estimated from the peak areas of Gaussian smoothed extracted ion chromatograms obtained with Xcalibur (Thermo Fisher) using an *m*/*z* tolerance of 5 ppm. Monoisotopic *m*/*z* values used are 830.9336 (M + 2H)^2+^, 554.2916 (M + 3H)^3+^, and 894.9810 (M + 2H)^2+^, 596.9899 (M + 3H)^3+^ for the two methylated peptides, respectively, and 673.3620 (M + 2H)^2+^ and 449.2439 (M + 3H)^3+^ for the nonmethylated peptide. The two methylated tryptic peptides were identified by Proteome Discoverer from the fragmentation spectrum produced by CID. To aid the identification of the unmethylated peptide representing residues 75–85 of subunit NDUFS2, a synthetic version was obtained from Cambridge Research Biochemicals (Billingham, UK).

## RESULTS

### 

#### 

##### Bioinformatic Identification of Putative Methyltransferases in Mitochondria

**FIGURE 1.**
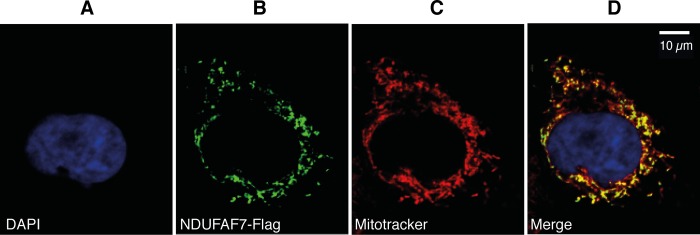
**Subcellular location of NDUFAF7.** Human 143B osteosarcoma cells were transfected with plasmid pcDNA^TM^5/FRT/TO containing the coding region for NDUFAF7 with C-terminal StrepII and FLAG tags and immunocytochemistry performed 24 h later. *A*, nucleus stained with DAPI (*blue*). *B*, recombinant NDUFAF7 visualized with anti-FLAG antibody and goat anti-mouse Alexa Fluor 488 (*green*). *C*, mitochondria stained with Mitotracker (*red*). *D*, *A–C* merged.

From among the 66 known and 142 putative human methyltransferases, 33 were predicted to have mitochondrial targeting sequences, including 23 representatives of the 7β-strand family of methyltransferases, five members of the family characterized by the presence of a SET domain, three spout methyltransferases, and two radical *S*-adenosylmethionine methylases (Table 1). Two additional proteins, NSUN3 and TRMT11, although not predicted to have processed N-terminal targeting sequences, nonetheless are known to be mitochondrial proteins (45). This analysis did not distinguish between protein and RNA methyltransferases. From among these 35 proteins, and taking into consideration the earlier work on MidA (30), NDUFAF7 was selected for study. Confirmation of its location in mitochondria was obtained by expressing the protein with a C-terminal FLAG tag in human osteosarcoma 143B cells and then by visualizing the protein with the aid of an antibody directed against the FLAG tag. Its location in the cells was coincident with mitochondria (Fig. 1) in confirmation of the bioinformatic prediction and of earlier observations (30).

**TABLE 1:** **Human mitochondrial methyltransferases** The table includes known mitochondrial methyltransferases and others identified by bioinformatic analysis. No attempt was made in the bioinformatic analysis to differentiate protein and RNA methyltransferases. The sequences of 66 methyltransferases of known function, and 142 putative methyltransferases of unknown function (36) were analyzed with MitoProt, iPSORT, and TargetP for the presence of N-terminal mitochondrial targeting sequences. Mitoprot scores of >0.5 indicate the presence of a targeting sequence. The family classification is taken from Ref. 36. The families of methyltransferases are as follows: 7β, 7β-strand; SET, SET domain (*Drosophila* Su(var)3–9, Enhancer of zeste and Trithorax); Spout, SpoU and TrmD methyltransferases; rad-Sam, radical *S*-adenosylmethionine. Cyt gra, cytoplasmic granules; ER, endoplasmic reticulum; hMito and yMito, human and yeast mitochondria; Mem, membranes.

Protein	Algorithm			Family	Function	Location
	MitoProt	iPSORT	TargetP			
ASMTL	0.58	−	+	7β	No	Unknown
C3orf51	0.88	+	+	7β	No	Unknown
COQ3*^a^*	0.99	+	+	7β	Yes (54)	hMito (45)
COQ5*^a^*	0.97	−	+	7β	Yes (55)	hMito (45)
FTSJ2*^b^*	0.48	−	+	7β	No (66)	Nucleus (66)
METTL7A	0.83	−	−	7β	No	ER, Mem (67, 68)
METTL8	0.79	−	+	7β	No	hMito (45)
METTL9	0.99	+	+	7β	No	Unknown
METTL12	0.96	-	+	7β	No	Unknown
METTL15	0.70	−	?	7β	No	hMito (45)
METTL17*^c^*	0.82	+	+	7β	No (69)	hMito (26)
METTL20	0.77	+	+	7β	No	Cyt gra (70)
NDUFAF5	0.99	+	+	7β	No	hMito (27)
NDUFAF7	0.96	+	+	7β	No	hMito (30)
NSUN3	0.12	−	?	7β	No	hMito (45)
NSUN4*^d^*	0.69	−	+	7β	No (71)	hMito (71)
PRMT8	0.73	−	+	7β	Yes (72)	Mem (72)
RRNAD1	0.58	+	+	7β	No	Unknown
TFB1M*^e^*	0.86	+	+	7β	Yes (73)	hMito (73)
TFB2M*^e^*	0.67	+	+	7β	Yes (74)	hMito (74)
TRMT1*^f^*	0.91	+	+	7β	Yes (75)	Unknown
TRMT2B	0.92	+	+	7β	No	hMito (26)
TRMT5*^g^*	0.66	−	+	7β	Yes (76)	Unknown
TRMT11	0.31	−	−	7β	No	hMito (45)
TRMT61B	0.74	−	+	7β	Yes (77)	hMito (45, 77)
EZHI*^h^*	0.46	+	?	SET	Yes (78)	Nucleus (78)
MLL*^h^*	0.78	+	?	SET	Yes (79)	Nucleus (80)
SETD9	0.99	+	+	SET	No	hMito (45)
WBP7*^h^*^,^*^i^*	0.64	−	?	SET	No (81)	Unknown
ZFPM1	0.99	+	+	SET	No	Unknown
MRM1*^j^*	0.99	−	+	Spout	No (82)	yMito (82)
RG9MTD1*^k^*	0.99	+	+	Spout	Yes (83)	hMito (83)
RNMTL1	0.93	+	+	Spout	No	hMito (26, 45)
CDK5RAP1	0.60	−	+	rad-SAM	No	Unknown
RSAD1	0.90	+	+	rad-SAM	No	Unknown

*^a^* Required for ubiquinone synthesis.

*^b^* rRNA methyltransferase in yeast.

*^c^* May be a component of the mitochondrial small ribosomal subunit.

*^d^* In the mitochondrial ribosome; forms a complex with MTERF4.

*^e^* Methylates mitochondrial 12 S rRNA.

*^f^* tRNA^Tyr^-methyltransferase.

*^g^* tRNA^Pro^-methyltransferase.

*^h^* Histone-lysine *N*-methyltransferase.

*^i^* Methyltransferase activity observed in mouse.

*^j^* Methylates yeast mitochondrial 21 S rRNA.

*^k^* Mitochondrial tRNA maturation.

##### Effect of Suppression of Mitochondrial NDUFAF7 on the Function of Complex I

**FIGURE 2.**
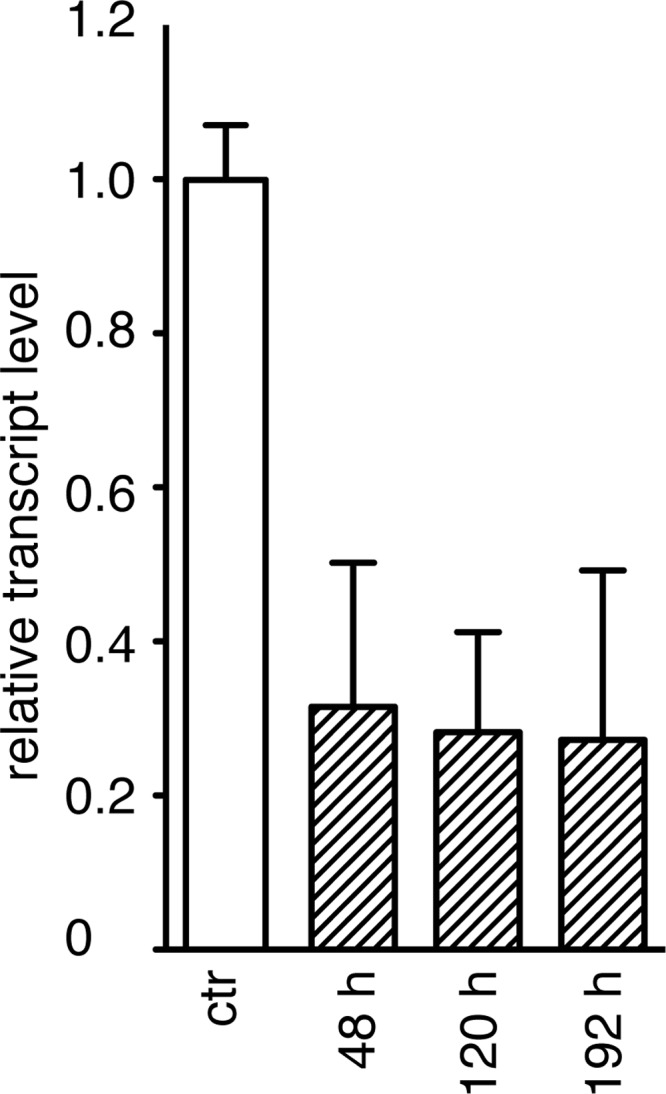
**Transient suppression of expression of NDUFAF7 in human 143B cells.** The cells were transfected three times at 72-h intervals with either siRNA directed against transcripts for NDUFAF7, or with control siRNA. The suppression of the transcript for NDUFAF7 was examined 48 h after each transfection (normalized to endogenous β-actin). The *hatched* and *white histograms* represent the levels of transcripts for NDUFAF7 and the control (*ctr*), respectively, and *error bars* show the standard deviation.

**FIGURE 3.**
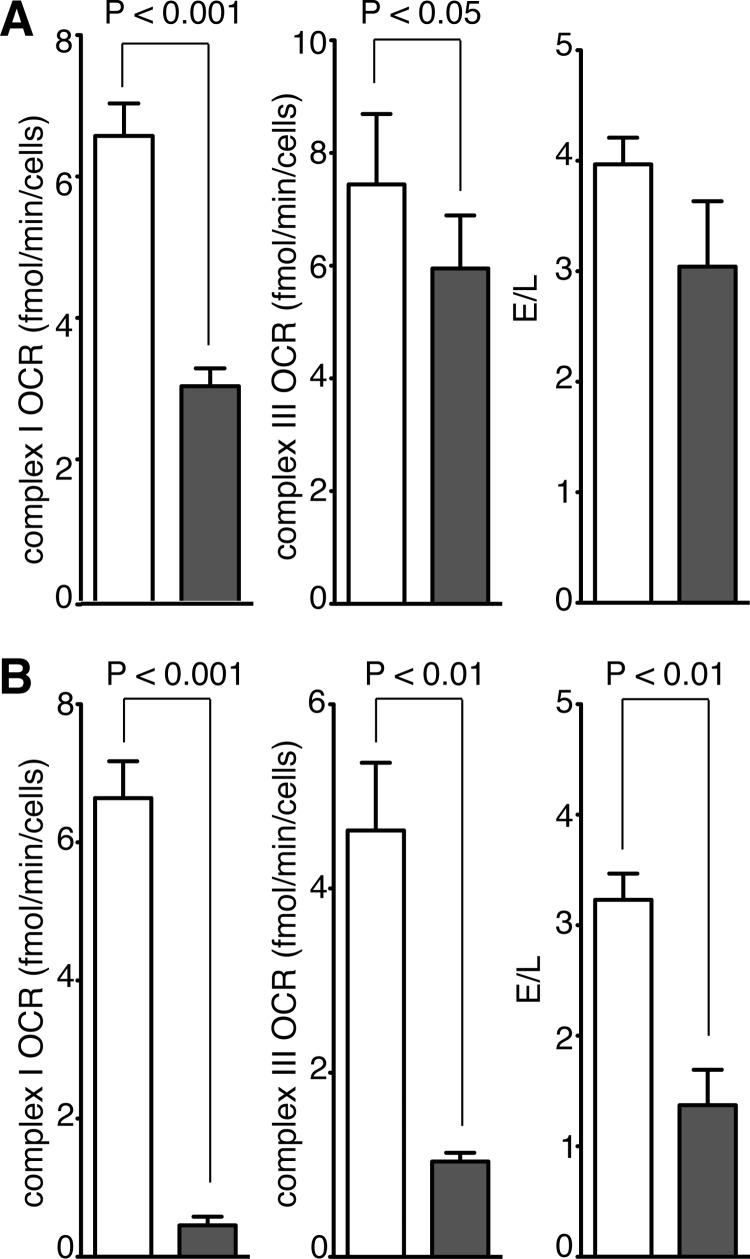
**Effect of transient suppression of expression of NDUFAF7 on oxygen consumption by human 143B cells.** Expression of NDUFAF7 was suppressed three times with siRNA at 72-h intervals and compared with the effect of control siRNA. The OCR at 120 and 192 h after the first transfection, normalized to cell number, was measured after the successive additions of 2-deoxyglucose, rotenone, duroquinol, and antimycin or 2-deoxyglucose, oligomycin, carbonylcyanide *p-*(trifluoromethoxy)phenylhydrazone, and a combination of rotenone and antimycin A. The *E*/*L* ratio (the carbonylcyanide *p-*(trifluoromethoxy)phenylhydrazone/oligomycin ratio) is an index of the maximum oxygen consumption capacity of the electron transport system (*E*) relative to the magnitude of the noncoupled respiration (*L*). *A* and *B* show the effect of suppression of expression of NDUFAF7 at 120 and 192 h, respectively. The *shaded* and *white bars* correspond to cells treated with siRNA against NDUFAF7 and control cells, respectively.

Transient suppression of expression of NDUFAF7 in human 143B cells decreased the level of transcripts for NDUFAF7 to ∼30% of the control (normalized to endogenous β-actin), and this level of suppression was maintained over the course of the experiment (Fig. 2). Associated with the suppression of expression of NDUFAF7, the cellular OCR linked to complex I was reduced by more than half after 120 h (Fig. 3*A*) and by more than 90% after 192 h (Fig. 3*B*). After 120 h, the reduction in OCR was confined mainly to complex I (Fig. 3*A*), but later, at 192 h, the effect on the OCR was more general, as shown by the large decreases in OCR linked to complex III and the *E*/*L* ratio (Fig. 3*B*; the E/L ratio is defined in the legend to Fig. 3). These experiments together with the bioinformatics analysis suggested that NDUFAF7 might be a protein methylase that modifies one of the subunits of complex I.

##### Effect of Suppression of Expression of NDUFAF7 on the Methylation of NDUFS2

**FIGURE 4.**
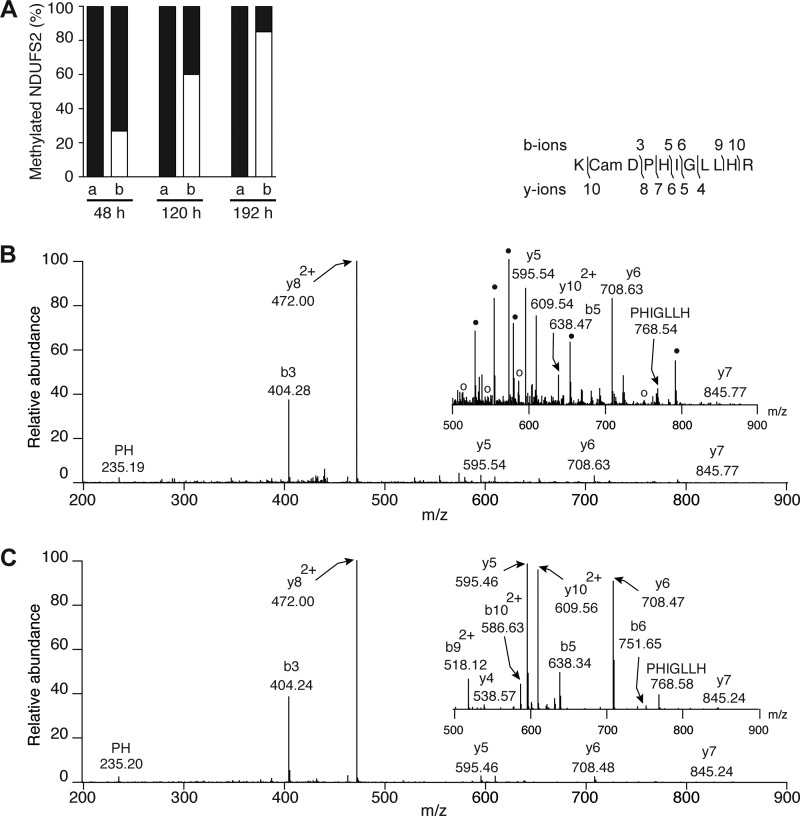
**Mass spectrometric analysis of the effect of suppression of expression of NDUFAF7 on the methylation status of Arg-85 in subunit NDUFS2 of complex I.** The 143B cells were transfected three times at 72-h intervals, with control siRNA, or with NDUFAF7 specific siRNA. The methylation status of tryptic peptides containing Arg-85 was examined by mass spectrometric analysis of tryptic digests of mitoplasts prepared from control and suppressed cells at 48, 120, and 192 h after the first transfection. In *A*, the histograms are derived from the extracted ion chromatograms for the *m*/*z* values of all possible methylation states for tryptic peptides containing Arg-85. Histograms labeled *a* and *b* correspond to control and suppressed cells, respectively. *Black* and *white areas* correspond to dimethylated and nonmethylated Arg-85, respectively. *B* and *C*, CID fragmentation spectra of, respectively, the tryptic peptide (residues 75–85 of NDUFS2) containing unmethylated Arg-85 derived from mitoplasts from cells where expression of NDUFAF7 has been suppressed and of a synthetic peptide with the same sequence KCamDPHIGLLHR. The *inset spectra* show a magnified *y* axis view from 500 to 900 *m*/*z*. In the *inset* in *B*, ○ denotes the ions b9^2+^ (517.97), y4 (538.46), b10^2+^ (586.57), and b6 (751.52) of the nonmethylated NDUFS2 peptide. The origin of the additional minor ions (●) is unknown. The *sequence inset* shows the fragment ions mapped onto the amino acid sequence.

Because Arg-85 in the NDUFS2 subunit of human complex I is dimethylated symmetrically and completely (37), the effect of suppression of expression of NDUFAF7 on the methylation status of this residue was investigated. In the tryptic digests of mitoplast proteins from control cells, the region of sequence of NDUFS2 containing Arg-85 was represented by two overlapping peptides, residues 75–89 and 76–89, in which Arg-85 remained fully dimethylated (Fig. 4). As found previously (37), there was no evidence of tryptic cleavage of the amide linkage between residues Arg-85 and Gly-86, as would be expected if this Arg-85 is fully dimethylated, and there was no evidence of monomethylated Arg-85. However, in the tryptic digests of the samples produced by transient suppression of expression of NDUFAF7, from 48 h, there was a progressive shift from dimethylated to nonmethylated status. At 120 h, only 40% of the peptide was dimethylated, and at 192 h the level of dimethylation had decreased further to 15%. Concomitant with these changes, increasing amounts of the unmethylated peptide (residues 75–85), arising from tryptic cleavage following the unmethylated Arg-85, were observed over the course of the experiment (26.9, 60, and 85%, respectively, at 48, 120, and 192 h; Fig. 4*A*). The identification of the unmethylated peptide by tandem MS was aided by comparison with the spectrum of a synthetic version of the same peptide (Fig. 4, *B* and *C*). The dominant ions in the CID spectra are common to both samples, arising from predominant cleavage at the Asp-Pro bond (b3 and y8 ions). The origin of the additional fragment ions in the magnified portion of the spectrum from the mitoplast sample (Fig. 4*B*, *inset*) is unknown, but they are likely to arise from minor contaminating tryptic peptides.

Hence, suppression of expression of NDUFAF7 is accompanied by suppression of dimethylation of Arg-85 of NDUFS2. Therefore, NDUFAF7 is a protein methylase that modifies residue Arg-85 of subunit NDUFS2 of complex I producing a symmetrically dimethylated guanidino group.

##### Effect of Suppression of NDUFAF7 on the Assembly of Complex I

**FIGURE 5.**
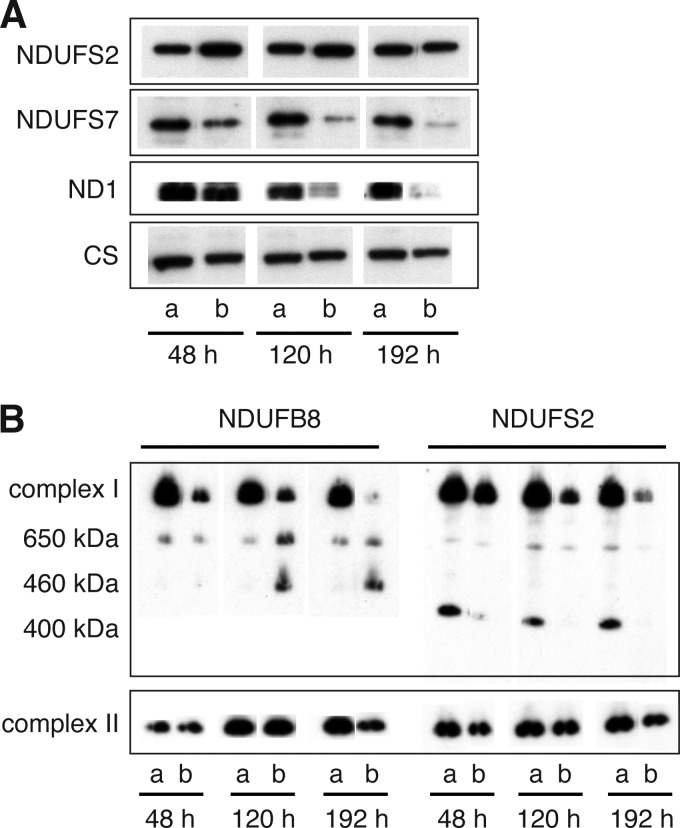
**Effect of transient suppression of expression of NDUFAF7 on the assembly of complex I.** Samples were prepared at the times indicated from human 143B cells that had been transfected three times with either control siRNA or siRNA specific for NDUFAF7 at 72-h intervals. In *A* and *B*, control and suppressed samples are denoted by *a* and *b*, respectively. *A*, the mitoplasts were fractionated by SDS-PAGE, blotted, and detected with antibodies against subunits NDUFS2, NDUFS7, and ND1 of complex I and with citrate synthase (*CS*) as a loading control. *B*, inner mitochondrial membrane proteins were fractionated by blue native PAGE, blotted, and detected with antibodies against NDUFB8 and NDUFS2, subunits in the membrane and peripheral arms of complex I, respectively, with an antibody against complex II as loading control.

To examine whether the suppression of methylation of Arg-85 affected the assembly of complex I in human 143B cells, respiratory complexes were fractionated by SDS-PAGE and blue native PAGE, and the integrity of complex I was examined by Western blotting. As shown in Fig. 5*A*, the level of NDUFS2 was unchanged over the course of the experiment. In contrast, NDUFS7, another peripheral arm subunit of complex I, and ND1, a mitochondrially encoded subunit in the membrane arm of complex I, were both lost progressively in NDUFAF7-depleted cells compared with control cells. This observation suggested that suppression of the methylation of NDUFS2 was influencing the assembly of complex I. This suggestion was confirmed by examination of complex I in cells in which expression of NDUFAF7 was suppressed where it was found that the level of intact complex I was reduced significantly relative to control cells (Fig. 5*B*). This reduction of intact complex I was accompanied by the accumulation of a subcomplex with an apparent molecular mass of 460 kDa, containing subunit NDUFB8, a component of the membrane arm of complex I, and by the loss of a subcomplex with an apparent molecular mass of 400 kDa containing subunit NDUFS2, a component of the peripheral arm of the enzyme.

## DISCUSSION

### 

#### 

##### Methylation of Proteins in Mitochondria

The *N*-methylation of proteins involves the enzymic addition of methyl groups donated by *S*-adenosylmethionine to the side chain nitrogen atoms of lysine and arginine, or more unusually to the imidazole side chain of histidine residues or to the α-N atom of the protein. Up to three methyl groups can be added to the ϵ-amino group of lysine, and the modified arginines, ω-*N*^G^-monomethylarginine, asymmetric ω-*N*^G^,*N*^G^-dimethylarginine and symmetric ω-*N*^G^,*N*^G′^-dimethylarginine, have all been observed. More and more mammalian proteins are being found to be methylated (46–48), and, among others, protein methylation has roles in regulating signal transduction, transcription, RNA processing, translation, DNA repair, protein translocation, protein interactions, and protein dynamics (46, 49, 50). Because lysine side chains can be modified also by acetylation and ubiquitination, the interplay between different modifications at the same site and adjacent sites can influence the functions of the proteins. Moreover, the actions of protein methylases and demethylases at the same site provide additional complexity in the role that methylation plays in the regulation of cellular activities (51–53). In the current work, 33 putative and known methyltransferases (22 putative, 11 known), including NDUFAF7, were identified by bioinformatic analysis as having the characteristics of proteins that are imported into mitochondria, plus NSUN3 and TRMT11, that are *bona fide* mitochondrial protein methylases (45). This list contains seven known RNA methylases because no attempt was made to distinguish between protein and RNA methylases (Table 1) and two proteins that participate in the biosynthesis of ubiquinone (54, 55). So far no protein demethylase has been identified in mitochondria either experimentally or by bioinformatics.

A small subset of proteins in mammalian mitochondria is known to be trimethylated completely and stably on the ϵ-amino group of specific lysine residues. They are citrate synthase, ADP-ATP translocase, and the c-subunit of ATP synthase, but the biological significance of these modifications remains unknown (56–58). Moreover, the mutation of yeast methyltransferase Ctm1p (59), the cytosolic enzyme responsible for modifying cytochrome *c*, had no impact on either the activity of cytochrome *c* or cell viability (60), and methylation is not essential for the activity of pig citrate synthase (61). In addition, two subunits of respiratory complex I are modified post-translationally by methylation: the NDUFB3 subunit has a complex pattern of methylation on three histidine residues near its N terminus (62), and Arg-85 of subunit NDUFS2, the subject of the current investigation, is symmetrically dimethylated (37).

In proteomic studies of mitochondria of trypanosomes, 167 proteins have been reported to be methylated (63), but in proteomic studies of methylated sites in mammalian cells, many fewer have been detected so far. In addition to the five mentioned already, only kynurenine-oxoglutarate transaminase 3, DNA-directed RNA polymerase, 60-kDa heat shock protein, serine-pyruvate aminotransferase, carbamoyl-phosphate synthase, prohibitin, hydroxymethylglutaryl-CoA synthase, polyribonucleotide nucleotidyltransferase 1, and 39 S ribosomal protein L35 are reported to be methylated (46–48) and present in both the MitoCarta and IMPI mitochondrial protein databases (26, 64). Among them, only kynurenine-oxoglutarate transaminase 3 is methylated on an arginine residue; the rest contain methylated lysine residues.

##### Role of NDUFAF7 as a Protein Methylase That Modifies Complex I

Until the present work, no methylase that modifies a protein in mammalian mitochondria had been identified unambiguously. In the experiments described above, it has been confirmed that NDUFAF7 localizes to the mitochondria of human 143B cells and that suppression of its expression has no influence on the level of NDUFS2, but that the methylation status of Arg-85 is progressively reduced with time, down to 15% after 192 h of suppression of expression. These experiments establish that NDUFAF7 is the enzyme in mitochondria that is responsible for the symmetrical dimethylation of Arg-85 of the NDUFS2 subunit of complex I, either during emergence into the mitochondrial matrix from the TIM complex involved in the import process and found in the inner membrane or following the release of the mature protein from the TIM complex into the matrix space. Protein arginine methyltransferases have been placed into three classes known as types I–III. All three types are associated with the formation of ω-*N*^G^-monomethylarginine, and the type I enzymes convert it to asymmetric ω-*N*^G^,*N*^G^-dimethylarginine. Only the type II enzymes produce symmetrical ω-*N*^G^,*N*^G′^-dimethylarginine, and there was no evidence for the formation of intermediate monomethylarginine in the current experiments. Therefore, NDUFAF7 belongs to type II of the arginine methyltransferases. The interaction of NDUFAF7 with NDUFS2 appears to be transient because no stable complex involving the two proteins was detected by examination of possible protein associations between the two proteins (data not shown).

##### Role of NDUFAF7 in the Assembly of Complex I

**FIGURE 6.**
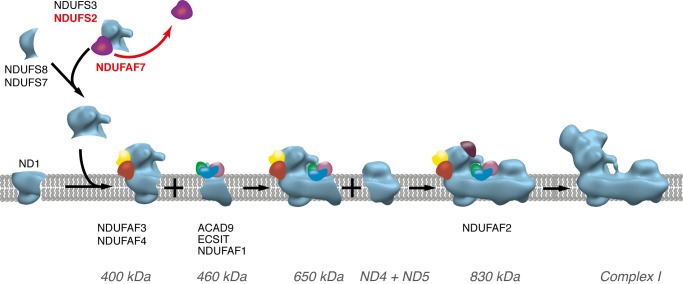
**Pathway of the assembly of complex I.** Methylation of NDUFS2 by NDUFAF7 occurs at an early step of assembly of complex I, before the formation of the 400-kDa subcomplex. It is probably required to stabilize the interaction with subunit NDUFS7, leading to the assembly of the 400-kDa subcomplex. The figure is based on Refs. 24 and 65.

The reduction in the activity of complex I (Fig. 3) and the loss of subunits NDUFS7 and ND1, both effects being concomitant with the suppression of expression of NDUFAF7, provided direct evidence that the methylation of Arg-85 in subunit NDUFS2 was required for the assembly of active complex I. The effect of partially blocking this methylation reaction on the integrity of the complex was evident from the analysis of complex I and related subcomplexes by blue native PAGE (Fig. 5). This analysis showed a reduction in the level of fully assembled complex I and of a related 400-kDa complex and concomitantly the accumulation of a 460-kDa subcomplex of complex I. Both the 400- and 460-kDa subcomplexes have been identified previously as intermediates in the pathway of assembly of complex I (Fig. 6). The 400-kDa subcomplex is an early assembly intermediate that, in addition to NDUFS2, contains the core catalytic subunits NDUFS3, NDUFS7, and NDUFS8 and the membrane subunit ND1 (16, 24). This subcomplex is thought to provide the membrane-proximal portion of the peripheral arm and the adjoining region of the membrane arm. In addition to NDUFS2, the membrane-proximal part of the peripheral arm contains subunit NDUFS7 and the covalently associated 4Fe-4S cluster, N2. Thus, it appears that the methylation of Arg-85 in NDUFS2 is required for the formation of the 400-kDa assembly intermediate. In the absence of the methylation, the subcomplex is not formed, and NDUFS7 and ND1 protein levels are depleted (Fig. 6), probably by the rapid turnover of newly synthesized proteins, similar to the effects of suppressing other assembly factors (NDUFAF3–6) involved in the formation of the 400-kDa intermediate (35). However, the levels of transcripts for ND1, NDUFS2, and NDUFS7 were unchanged by suppression of expression of NDUFAF7 (data not shown). NDUFAF5, another complex I assembly factor and a putative methyltransferase, is also required for the stable formation of the initial peripheral arm subcomplex (27, 35). Although suppression of expression of NDUFAF5 disrupted the assembly of the 400-kDa intermediate, there was no associated change in the methylation status of NDUFS2 (data not shown). Pathogenic mutations are associated with NDUFAF5, and also with many, but not all, of the proteins that assist in the assembly of complex (20, 23, 25, 29, 32, 33). So far, none has been found to be associated with NDUFAF7. Because NDUFAF7 acts early in the pathway of assembly of complex I, human mutations affecting its function may be lethal.

**FIGURE 7.**
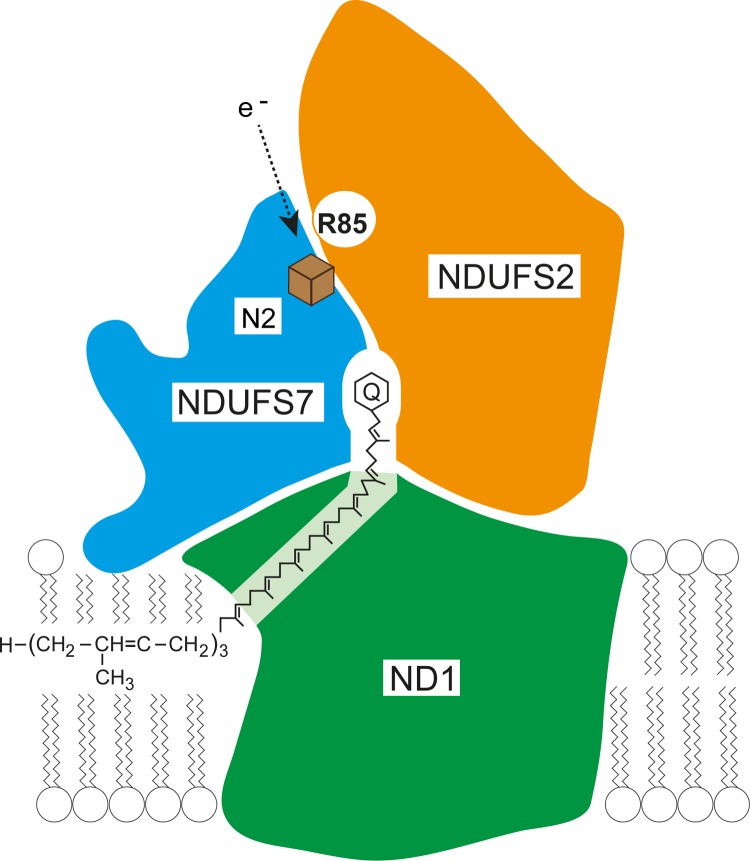
**Location of dimethylated Arg-85 in the vicinity of the quinone binding site of human complex I.** The outlines of the shapes of the NDUFS2, NDUFS7, and ND1 subunits are shown in *orange*, *blue*, and *green*, respectively; the *green* ND1 subunit is divided by the intervening quinone binding site into two unequal areas: one small and triangular and the other larger and pentangular. The area shaded *lighter green* indicates that the two *dark green areas* are joined in front and behind the quinone site. The scheme is based upon the structures and positional relationships of the orthologous core proteins Nqo4, Nqo6, and Nqo8 in the structure of complex I from *T. thermophilus* (6). The view is along the axis of the membrane arm of complex I away from its junction with the orthologous extrinsic arm (vertical pointing upwards into the matrix of the mitochondrion). The approximate position of the phospholipid bilayer of the inner mitochondrial membrane is indicated. The quinone is shown with its head group and part of its side chain bound in a tunnel between the three subunits. The entrance to the tunnel is formed by the transmembrane α-helices 1 and 6 and amphipathic α-helix 1 of subunit Nqo8. The dimethylated Arg-85 in the human NDUFS2 subunit is in a loop between β-strand 3 and α-helix 1 close to iron-sulfur cluster N2 (*brown cube*), which is attached to the NDUFS7 subunit. The *arrow* indicates the direction of electron flow from the penultimate iron-sulfur cluster to the terminal cluster, N2. The figure is adapted from Carroll *et al.* (37).

It is possible that the methylation of Arg-85 in the NDUFS2 subunit of complex I plays a role not only in the assembly of the complex, but also in the mechanism of the enzyme. In the structure of complex I from *Thermus thermophilus*, the orthologues of human subunits NDUFS2 and NDUFS7, together with subunit ND1, provide the binding pocket for ubiquinone (6), and it is most likely that the human orthologues have a similar disposition (Fig. 7) and also provide the binding site for the quinone. Subunit NDUFS7 contains covalently bound iron-sulfur cluster N2, which is the terminal iron-sulfur cluster in the chain of seven iron-sulfur clusters linking a noncovalently bound FMN molecule, the primary acceptor of electrons from NADH, to the terminal electron acceptor, ubiquinone. In this model, methylated Arg-85 of subunit NDUFS2 by analogy with the position of the orthologous threonine residue 64 in the structure of complex I from *T. thermophilus* is ∼7 Å (or possibly even closer) from iron-sulfur cluster N2 (Fig. 7). The methylation of the side chain of this residue has a number of effects. It increases its hydrophobicity and the solvent-accessible area of the side chain, and reduces its potential to form hydrogen bonds. Methylation also lowers its pI value slightly, and so it might conceivably influence the redox potential of cluster N2. The evaluation of this possibility must probably await an atomic structure of mammalian complex I.

## Footnotes

3 The abbreviations used are:

OCR: rate of consumption of oxygen
CID: collision-induced dissociation
Cam: carbamidomethylcysteine.
